# The mediating role of personality traits in the relationship between challenge threat appraisal and pro-antisocial behaviors

**DOI:** 10.3389/fpsyg.2026.1797673

**Published:** 2026-04-10

**Authors:** Samet Sitti, Suna Dincel, Serdar Solmaz, Yunus Emre Yarayan, Berzan Şimşek

**Affiliations:** 1 Department of Movement and Training Sciences, Siirt Universitesi, Siirt, Türkiye; 2 Department of Physical Education and Sports Teaching, Gazi Universitesi, Ankara, Türkiye; 3 Department of Sports Management, Batman University, Batman, Türkiye

**Keywords:** antisocial behavior, challenge appraisal, extraversion, neuroticism, prosocial behavior, threat appraisal

## Abstract

**Background:**

Modern football performance is a multifaceted phenomenon where cognitive-emotional appraisals directly influence on-field interpersonal behaviors. This study adopts a holistic approach to examine how challenge and threat appraisals affect professional football players’ prosocial and antisocial behaviors. Specifically, it investigates the mediating role of extraversion and neuroticism in transforming these cognitive perceptions into competitive social actions, aiming to provide actionable insights for coaches to optimize players’ behavioral performance during high-pressure match scenarios.

**Methods:**

The research sample consisted of 601 professional football players who were actively engaged in the football disciplines, including 279 women (%46.4 *M*_age_ = 21.37, SD = 3.38) and 322 men (%53.6 *M*_age_ = 21.35, SD = 2.72). Data were collected using the Challenge and Threat in Sport Scale, the Prosocial and Antisocial Behavior in Sport Scale, and the Big Five Personality Inventory (extraversion and neuroticism subscales). After testing normality assumptions, path analysis was performed using R software to assess both direct and indirect effects within a competitive soccer context.

**Results:**

Path analysis revealed that challenge appraisal is a significant positive predictor of prosocial behavior and a negative predictor of antisocial behavior. While threat appraisal directly increased antisocial tendencies potentially compromising tactical discipline it only influenced prosocial behavior indirectly through personality traits. Specifically, extraversion significantly mediated the link between challenge appraisal and prosocial actions, while neuroticism mediated the impact of threat appraisal on prosocial outcomes. The model accounted for a modest proportion of variance in both prosocial (9.6%) and antisocial (5.8%) behaviors, suggesting that personality traits and cognitive appraisals are meaningfully associated with social behaviors in sport contexts.

**Conclusion:**

These findings underscore that optimizing football performance requires more than physical or tactical training; it demands psychological resilience. Fostering a challenge-oriented mindset and leveraging personality-based resources can mitigate antisocial behaviors that lead to disciplinary issues and enhance prosocial interactions that strengthen team cohesion. For technical staff, these results suggest that psychological profiling and cognitive reframing interventions are essential tools for programming effective training sessions and improving player behavior during competition.

## Introduction

Challenge and threat perceptions are among the key factors shaping individuals’ social interactions and behaviors (Blascovich and Mendes, 2000). These appraisals are closely associated with how individuals respond to difficulties and whether their reactions take the form of prosocial or antisocial behaviors. The emergence of social behavior is a complex process shaped by the interaction between personal traits and environmental conditions. Examining prosocial and antisocial behaviors within the context of moral tendencies and social dynamics offers valuable insights into individuals’ social relationships (Eisenberg and Fabes, 1998; Kavussanu, 2006). The sports environment provides a unique context for shaping social relationships and testing psychological resilience. Elements such as team cohesion, fair play, and leadership offer a meaningful basis for evaluating athletes’ social and moral capacities (Shields and Bredemeier, 1995). Accordingly, sport psychology research provides important data for understanding how individuals develop in their social interactions.

Athletes behaviors are shaped not only by individual traits but also by the complex influences of group dynamics and environmental interactions (Al-Yaaribi and Kavussanu, 2017). The impact of challenge and threat appraisals on athletes is closely linked to their personality traits (Çelikten and Demirli, 2018). Prosocial behaviors, in particular, promote team cooperation, enhancing social cohesion and a sense of satisfaction. For example, helping an opponent or encouraging a teammate plays a key role in strengthening social bonds (Al-Yaaribi et al., 2016). In contrast, antisocial behaviors foster conflict, increase burnout levels, and negatively affect performance (Micai et al., 2015). Therefore, investigating the determinants of prosocial and antisocial behaviors in sport is essential for gaining a deeper understanding of social interactions in athletic contexts (Kavussanu and Boardley, 2009).

Research aimed at understanding the influence of personality traits on social behaviors highlights the complex nature of these dynamics. The Five-Factor Model of Personality offers a comprehensive framework for explaining behavioral patterns in social interactions (McCrae and Costa, 1989). This model includes five core dimensions extraversion, agreeableness, conscientiousness, neuroticism, and openness to experience, each of which may play a mediating role in shaping individuals’ attitudes and behaviors in social contexts. Extraversion promotes effective communication and collaboration in social settings, while agreeableness enhances empathy and the tendency to help others (Graziano et al., 2007). Individuals high in agreeableness tend to provide greater support in social relationships, strengthening cooperation within groups. Conversely, low levels of agreeableness are associated with a greater likelihood of antisocial behaviors (Graziano et al., 2007). Neuroticism is a key predictor of individuals’ resilience in the face of stress and anxiety (McCrae and Costa, 1989). High neuroticism is generally linked to increased threat perception and social withdrawal, whereas low neuroticism is associated with more effective social engagement (Lahey, 2009). Especially in high-stress environments like sports, this trait plays a critical role in athletes’ psychological resilience and social adjustment (Fletcher and Sarkar, 2012). Additionally, openness to experience is associated with adaptability, creativity, and strategic thinking in dynamic sport environments (Feist, 1998). This trait may foster prosocial behaviors by promoting team cooperation and contributing to the development of collective awareness (John and Srivastava, 1999). Overall, personality traits provide a fundamental framework for understanding individuals’ social relationships and team dynamics.

Beyond personality traits, challenge and threat appraisals are important factors associated with individuals’ social behaviors. Challenge appraisal arises in individuals who perceive stressful situations as opportunities and is typically associated with positive emotions (Jones et al., 2009; Rossato et al., 2018). This perception encourages active problem-solving in the face of adversity and is directly related to resilience (Tenenbaum and Eklund, 2001). Individuals with high levels of challenge appraisal tend to approach stressful situations with a positive outlook, which may enhance their social relationships and increase prosocial behaviors. Acting with positive emotions may further facilitate prosocial tendencies (Jones et al., 2009). In contrast, threat appraisal emerges when individuals believe they lack sufficient resources to effectively manage a given situation and is commonly associated with anxiety and stress (Blascovich and Mendes, 2000). Those with high threat appraisal may adopt a defensive attitude in social interactions, potentially diminishing their prosocial behaviors (Lazarus and Folkman, 1984). Moreover, threat appraisal has been associated with lower stress-coping abilities and disruptions in social relationships. In sum, challenge and threat appraisals influence social dynamics in divergent ways. While challenge appraisal supports the development of positive coping strategies and strengthens social bonds, threat appraisal shapes interactions in a more defensive and adverse direction. These two cognitive appraisals appear to be associated with social behavior in different ways.

Research examining the effects of threat appraisal on individuals’ behavioral responses suggests that this perception interacts with personality traits (Carver and Connor-Smith, 2010; Lazarus and Folkman, 1984). In this context, the influence of threat appraisal on prosocial and antisocial behaviors becomes more complex when considered through the lens of personality traits. Personality plays a critical role in shaping individuals’ social interactions. For instance, individuals with high levels of empathy tend to respond more constructively to threat appraisals, which can positively influence group dynamics (Joyal-Desmarais et al., 2024). Conversely, threat appraisal among individuals high in agreeableness has been associated with a greater likelihood of social conflict, potentially undermining interpersonal relationships (Stephan and Stephan, 2000). Studies in this area provide a valuable foundation for better understanding psychological processes and social interactions, particularly within disciplines such as sport psychology.

In this context, personality traits offer valuable insights into the relationship between challenge and threat appraisals and social behaviors. Positive personality characteristics such as conscientiousness and extraversion tend to enhance challenge appraisal while reducing threat appraisal (Basım et al., 2009). Individuals with these traits are more likely to cope effectively with stress and to reframe threatening situations as challenges. In particular, extraverted individuals tend to take an active role in social interactions and adopt a proactive approach toward environmental stressors. However, high levels of neuroticism are associated with an increased tendency toward threat appraisal, which may, in turn, lead to antisocial behaviors (McCrae and Costa, 1989). Neuroticism not only influences how individuals respond to stress but can also result in negative social outcomes and pose a risk to an individual’s psychological well-being.

The decisive role of individual differences in shaping the dynamics of social interaction holds a prominent place in the sport psychology literature (Diamant et al., 2019). Research findings support that positive personality traits such as extraversion and conscientiousness enhance challenge appraisal and reduce threat appraisal, whereas neuroticism increases threat appraisal and creates a predisposition toward antisocial behaviors. In this regard, the present study aims to examine the effects of challenge and threat appraisals on individuals’ social behaviors while analyzing the mediating role of personality traits specifically neuroticism and extraversion in these relationships. Developing a deeper understanding of how challenge and threat perceptions shape individuals’ tendencies toward prosocial and antisocial behaviors is expected to contribute to the field of sport psychology at both theoretical and applied levels.

## Method

### Research design

This study was designed within the framework of a correlational survey model to examine the direct and indirect relationships among the variables. Specifically, the effects of threat and challenge appraisals on prosocial and antisocial behaviors were analyzed, along with the mediating role of personality traits (extraversion and neuroticism), using path analysis. Path analysis is a statistical technique that allows researchers to examine structural and predictive relationships among variables, enabling the evaluation of both direct and indirect effects (Kline, 2015).

### Participants

The study sample consisted of 601 professional football players who were actively engaged in the football disciplines, including 279 women (%46.4, *M*_age_ = 21.37, *SD* = 3.38) and 322 men (%53.6, *M*_age_ = 21.35, *SD* = 2.72). The average number of years of athletic experience was 4.60 years (*SD* = 2.59) for female participants and 5.78 years (*SD* = 3.48) for male participants. The sample size meets the minimum recommended criteria for participant-to-variable ratios in social sciences (Tabachnick and Fidell, 2019) and satisfies the statistical power requirements emphasized by Cohen (1992).

### Inclusion and exclusion criteria

Participants were included in the study if they were actively competing as professional football players at the time of data collection, voluntarily agreed to participate, and fully completed all measurement instruments. Only athletes who met these criteria were deemed eligible for inclusion. Players were excluded if they were not currently engaged in professional football, declined voluntary participation, or provided incomplete, inconsistent, or invalid responses (e.g., patterned responses, excessive missing data). Additionally, athletes who did not meet the minimum requirements for participation (such as being officially registered with a professional club) were excluded to ensure accuracy and homogeneity.

### Ethical considerations

This study was conducted in accordance with ethical principles and guidelines. The research protocol was reviewed and approved by the Ethics Committee of Siirt University during its 566th meeting held on April 11, 2023, confirming that the study complied with ethical standards.

### Data collection tools

#### Personal information form

The form developed by the researchers includes questions regarding participants gender, age, sport discipline, and years of athletic experience.

#### Challenge and threat in sport scale

In this study, the Challenge and Threat in Sport Scale (CTSS), developed by Rossato et al. (2018), was used to assess athletes’ perceptions of challenge and threat. The scale consists of 12 items designed to measure perceptions of challenge and threat in the sports context and includes two subdimensions: challenge and threat. The CTSS is a 6-point Likert-type scale, where participants rate each item from 1 (strongly disagree) to 6 (strongly agree). The Turkish adaptation of the scale was conducted by Türkyilmaz and Altıntaş (2020), and its validity and reliability were tested on a sample of professional football players. Results of exploratory and confirmatory factor analyses confirmed the two-factor structure of the scale in the Turkish sample. The Cronbach’s alpha coefficients for the subscales were calculated as 0.81 for the challenge subscale and 0.82 for the threat subscale, indicating good internal consistency. In the current study, Cronbach’s alpha coefficients were found to be 0.79 for the challenge subscale and 0.75 for the threat subscale.

#### Prosocial and antisocial behavior in sport scale

In this study, the Prosocial and Antisocial Behavior in Sport Scale (PABSS), developed by Kavussanu and Boardley (2009), was used to assess athletes prosocial and antisocial behaviors. The Turkish adaptation of the scale was conducted by Balcikanli (2013). The scale consists of 20 items and four subdimensions designed to measure prosocial and antisocial behaviors in the context of sport. It is a 5-point Likert-type scale on which participants are asked to rate each item from 1 (never) to 5 (very often). The Cronbach’s alpha reliability coefficients for the subdimensions in the Turkish adaptation were found to be 0.70, 0.72, 0.72, and 0.75, indicating that the scale is reliable. In the present study, the subdimensions were combined into two main categories: general prosocial behaviors and general antisocial behaviors. This approach was adopted to evaluate athletes’ behavioral tendencies from a broader perspective, in line with the overall aim of the study. Rather than focusing on distinctions between behaviors directed toward opponents and teammates, the goal was to capture athletes’ general behavioral inclinations. In this study, the internal consistency coefficients were found to be 0.72 for prosocial behaviors and 0.86 for antisocial behaviors.

#### Big five personality inventory

In this study, the Big Five Inventory (BFI), developed by Rammstedt and John (2007), was used to assess participants’ personality traits. The scale evaluates personality across five major dimensions: extraversion, neuroticism, openness to experience, agreeableness, and conscientiousness. The Turkish adaptation was conducted by Horzum et al. (2017). The scale consists of 10 items, with two items measuring each personality dimension. It is a 5-point Likert-type scale, and participants are asked to rate each item from 1 (never) to 5 (always). Exploratory and confirmatory factor analyses confirmed the validity of the five-factor structure in the Turkish sample. The internal consistency coefficients (Cronbach’s alpha) for the subscales were found to be 0.88 for extraversion, 0.81 for agreeableness, 0.90 for conscientiousness, 0.85 for neuroticism, and 0.84 for openness to experience, indicating that the scale is a reliable measurement tool. In the present study, only the extraversion and neuroticism dimensions were included as mediating variables to better understand the effects of challenge and threat appraisals on social behaviors. These dimensions were selected based on existing literature indicating that extraversion supports prosocial behaviors, while neuroticism is associated with antisocial behaviors. In the current sample, the internal consistency coefficients were calculated as 0.81 for extraversion and 0.79 for neuroticism.

#### Data collection procedure

The data collection process of this study lasted approximately 3 months and was carried out using two different methods. Both online and face-to-face approaches were used to reach participants. For the online data collection, the study instruments were formatted via Google Forms and digitally distributed to participants. The online form was designed to facilitate professional football players access and to reach a broader sample. In addition, face-to-face data collection involved direct communication with football teams. During this process, the purpose of the study was explained in detail, and the scales were administered accordingly.

All data were collected on a voluntary basis, with strict attention paid to protecting participants’ privacy and personal information. Participants were informed that the study was conducted for scientific purposes and that their responses would be used solely for research-related analyses.

#### Data analysis and interpretation

Prior to conducting statistical analyses, data preparation was carried out meticulously. First, missing and incomplete data were examined. To enhance data accuracy, mean substitution was applied for items with less than 5% missing data. However, eight participants with substantial missing responses were excluded from the analyses. Next, outlier analysis was conducted using Mahalanobis distance (Mahalanobis D^2^), a widely used method for identifying multivariate outliers in statistical analyses (Kline, 2015). Twelve participants with values exceeding the critical threshold were excluded on the grounds that they could distort the overall distribution and threaten the reliability of the results. This step was essential for ensuring data homogeneity and increasing model validity.

In the subsequent phase, multivariate normality assumptions were assessed. Skewness and kurtosis values were used as indicators of normality. Values falling within the range of −2 to +2 were accepted as evidence of normal distribution (George and Mallery, 2010). Based on these criteria, the data were determined to meet the assumptions of normality and were deemed suitable for multivariate analysis.

Following data preparation, Pearson correlation analysis was conducted to examine the relationships among the variables. To test both direct and indirect effects, path analysis was employed. All analyses were performed using R, an open-source statistical software. Data processing, statistical computations, and visualization of results were carried out through this platform. These procedures ensured accurate preparation, analysis, and interpretation of the data, thereby enhancing the reliability and validity of the findings.

## Findings

Table 1 presents the descriptive statistics for the variables included in the study. Skewness and kurtosis values were examined to assess the assumption of normality. According to George and Mallery (2010), skewness and kurtosis values between −2 and +2 are considered acceptable to satisfy the normality assumption.

**Table 1:** Descriptive statistics for the variables included in the study.

Variables	*N*	Mean	Sd	Skewness	Kurtosis
Challenge	601	21.424	5.910	−0.445	−0.245
Threat	601	20.353	7.180	0.144	−0.365
Extraversion	601	6.932	2.046	−0.347	−0.353
Neuroticism	601	5.667	1.963	0.070	−0.452
Prosocial behavior	601	25.649	5.804	−0.561	0.004
Antisocial behavior	601	31.328	11.193	0.435	−0.627

The correlation analysis results presented in Table 2 reveal the relationships among the study variables. A positive and significant relationship was found between challenge appraisal and prosocial behavior (*r* = 0.237, *p* < 0.01). Similarly, challenge appraisal was negatively and significantly associated with antisocial behavior (*r* = −0.172, *p* < 0.01). On the other hand, a positive and significant relationship was observed between threat appraisal and antisocial behavior (*r* = 0.135, *p* < 0.01). The absence of a significant relationship between threat appraisal and prosocial behavior (*r* = −0.095, *p* > 0.05) suggests that threat appraisal does not have a notable impact on positive social behaviors. Furthermore, a positive and significant correlation was found between extraversion and prosocial behavior (*r* = 0.172, *p* < 0.01), while a negative and significant correlation emerged between neuroticism and prosocial behavior (*r* = −0.185, *p* < 0.01). Lastly, the lack of a significant relationship between neuroticism and antisocial behavior (*r* = 0.082, *p* > 0.05) indicates that this personality trait is not distinctly associated with negative social behaviors. These analyses demonstrate the direction and strength of the relationships among variables, supporting the study’s theoretical framework. Based on these findings, path analysis was conducted, and the results are presented in Table 3.

**Table 2:** Correlation analysis results for the relationships among variables.

Variables	Mean	Sd	1	2	3	4	5	6
1. Challenge	21.42	5.91	1					
2. Threat	20.35	7.18	0.057	1				
3. Extraversion	6.93	2.05	0.181^**^	−0.210^**^	1			
4. Neuroticism	5.67	1.96	−0.074	0.218^**^	−0.164^**^	1		
5. Prosocial behavior	25.65	5.80	0.237^**^	−0.095^*^	0.172^**^	−0.185^**^	1	
6. Antisocial behavior	31.33	11.19	−0.172^**^	0.135^**^	0.000	0.082^*^	−0.097^*^	1

**p < 0.01, *p < 0.05.

**Table 3:** Findings from the path analysis.

	% 95 CI					
Variables	β	LL	UL	Std. Err	*z*-value	*P*(>|*z*|)
Direct effect						
Threat appraisal ➔ Prosocial	−0.05	−0.133	0.022	0.03	−1.40	0.16
Extraversion ➔ Prosocial	0.09*	0.021	0.176	0.04	2.48	0.01
Neuroticism ➔ Prosocial	−0.14^*^	−0.219	−0.062	0.04	−3.52	0.00
Challenge appraisal ➔ Prosocial	0.21	0.126	0.298	0.04	4.85	0.00
Threat appraisal ➔ Antisocial	0.15^**^	0.063	0.240	0.04	3.36	0.00
Extraversion ➔ Antisocial	0.07	−0.012	0.159	0.04	1.69	0.09
Neuroticism ➔ Antisocial	0.04	−0.036	0.130	0.04	1.11	0.26
Challenge appraisal ➔ Antisocial	−0.19^**^	−0.272	−0.110	0.04	−4.62	0.00
Threat appraisal ➔ Neuroticism	0.22^**^	0.143	0.304	0.04	5.42	0.00
Challenge appraisal ➔ Neuroticism	−0.08*	−0.162	−0.011	0.03	−2.24	0.02
Threat appraisal ➔ Extraversion	−0.22^**^	−0.304	−0.138	0.04	−5.19	0.00
Challenge appraisal ➔ Extraversion	0.19^**^	0.116	0.270	0.03	4.91	0.00
Mediator effect						
Challenge appraisal ➔ Neuroticism ➔ Prosocial	0.01	−0.001	0.025	0.00	1.82	0.06
Threat appraisal ➔ Neuroticism ➔ Prosocial	−0.03^**^	−0.052	−0.010	0.01	−2.93	0.00
Challenge appraisal ➔ Extraversion ➔ Prosocial	0.01^*^	0.003	0.035	0.00	2.28	0.02
Threat appraisal➔ Extraversion ➔ Prosocial	−0.02*	−0.042	−0.002	0.01	−2.14	0.03
Challenge appraisal ➔ Neuroticism ➔ Antisocial	−0.00	−0.012	0.004	0.00	−1.00	0.31
Threat appraisal ➔ Neuroticism ➔ Antisocial	0.01	−0.009	0.030	0.01	1.08	0.28
Challenge appraisal ➔ Extraversion ➔ Antisocial	0.01	−0.004	0.032	0.00	1.53	0.12
Threat appraisal extraversion ➔ Antisocial	−0.01	−0.036	0.004	0.01	−1.57	0.11

**p < 0.01, *p < 0.05, Std. Err, Standard Error; Standardized beta coefficients (β) are reported.

Table 3 presents a detailed overview of the relationships among threat and challenge appraisals, personality traits, and prosocial and antisocial behaviors.

When examining direct effects, threat appraisal was found to have a negative but statistically non-significant effect on prosocial behavior (β = −0.05, 95% CI: −0.133, 0.022; *p* = 0.16). This finding suggests that threat appraisal does not significantly influence individuals’ prosocial behaviors. In contrast, challenge appraisal had a positive and significant effect on prosocial behavior (β = 0.21, 95% CI: 0.126, 0.298; *p* = 0.00), indicating that perceiving stressful situations as challenges may promote positive social behaviors. Additionally, extraversion was positively and significantly associated with prosocial behavior (β = 0.09, 95% CI: 0.021, 0.176; *p* = 0.01), suggesting that individuals with higher levels of extraversion are more likely to exhibit prosocial behaviors. Neuroticism, on the other hand, had a negative and significant effect on prosocial behavior (β = −0.14, 95% CI: −0.219, −0.062; *p* = 0.00), indicating that higher levels of neurotic traits may suppress positive social behaviors.

In terms of antisocial behaviors, threat appraisal was found to have a positive and statistically significant effect (β = 0.15, 95% CI: 0.063, 0.240; *p* = 0.00). This finding indicates that individuals with higher levels of threat appraisal are more likely to engage in antisocial behaviors. Conversely, challenge appraisal negatively and significantly predicted antisocial behavior (β = −0.19, 95% CI: −0.272, −0.110; *p* = 0.00), suggesting that perceiving stressful situations as challenges may play a protective role by reducing negative social behaviors.

When examining the effects on personality traits, threat appraisal was found to have a positive and significant effect on neuroticism (β = 0.22, 95% CI: 0.143, 0.304; *p* = 0.00). Additionally, threat appraisal had a negative and significant effect on extraversion (β = −0.22, 95% CI: −0.304, −0.138; *p* = 0.00). These findings suggest that threat appraisal may influence different personality traits in opposite directions. Challenge appraisal, on the other hand, had a negative and significant effect on neuroticism (β = −0.08, 95% CI: −0.162, −0.011; *p* = 0.02), indicating that perceiving challenges may help reduce neurotic tendencies. Moreover, challenge appraisal had a positive and significant effect on extraversion (β = 0.19, 95% CI: 0.116, 0.270; *p* = 0.00), indicating a positive association between challenge appraisal and extraversion.

When examining the mediating effects, threat appraisal was found to negatively influence prosocial behavior through neuroticism (β = −0.03, 95% CI: −0.052, −0.010; *p* = 0.00). Similarly, threat appraisal also had a negative indirect effect on prosocial behavior through extraversion (β = −0.02, 95% CI: −0.042, −0.002; *p* = 0.03). On the other hand, challenge appraisal was found to positively influence prosocial behavior through extraversion (β = 0.01, 95% CI: 0.003, 0.035; *p* = 0.02).

**Figure 1:**
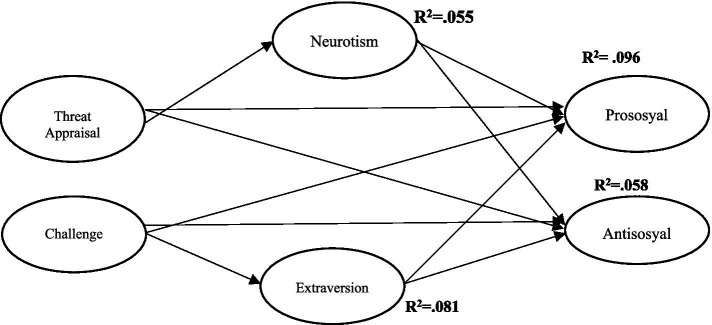
Path model.

These findings demonstrate that threat and challenge appraisals significantly influence individuals’ social behaviors, particularly through the mediating role of personality traits. While challenge appraisal appears to support prosocial behavior and reduce antisocial tendencies, threat appraisal may undermine individuals’ social adjustment. The mediating function of personality traits in this relationship provides a deeper understanding of how social behaviors are shaped by the interaction between personal and environmental factors. These results contribute to the literature by offering a clearer understanding of the links between personality traits, challenge/threat appraisals, and prosocial and antisocial behaviors. Moreover, they highlight the importance of implementing targeted interventions aimed at enhancing individuals’ behavioral outcomes. The path model developed as part of this study is presented in Figure 1.

## Discussion

This study examined the effects of threat and challenge appraisals on individuals’ prosocial and antisocial behaviors, as well as the mediating role of personality traits in these relationships. The findings revealed that threat appraisal did not have a significant direct effect on prosocial behavior, whereas challenge appraisal was positively associated with prosocial behavior. Additionally, threat appraisal was positively related to antisocial behavior, whereas challenge appraisal was negatively related to such behaviors. These direct effects are consistent with previous studies that explain how individuals’ cognitive and emotional evaluations of their environment are shaped by threat and challenge perceptions (Blascovich and Mendes, 2000; Deci and Ryan, 2000; Kavussanu and Boardley, 2009).

When examining the findings related to personality, it was observed that extraversion increased prosocial behaviors, whereas neuroticism decreased them. This may be due to the fact that extraverted individuals tend to have higher social awareness and are more inclined toward cooperative behaviors, which may lead them to associate challenge appraisals with more positive social outcomes (Ashton and Lee, 2007; Graziano et al., 2007). In contrast, neurotic individuals may respond to stressful or ambiguous social situations with anxiety and withdrawal, thereby linking threat appraisals to negative social behaviors (Eisenberg et al., 2000; Kokkonen and Pulkkinen, 2001). Furthermore, it was found that threat appraisal was positively associated with neuroticism and negatively associated with extraversion, whereas challenge appraisal was negatively associated with neuroticism and positively associated with extraversion. These results suggest that personality traits may vary across contexts depending on how individuals cognitively process threat and challenge appraisals. The literature includes studies that examine the relationship between threat/challenge appraisals and personality traits (Fletcher and Sarkar, 2012; Komulainen et al., 2014). Considering that the participants in this study were professional athletes, it may be argued that the positive effect of challenge appraisal on extraversion could be linked to the resilience fostered by the competitive nature of sports. The tendency of athletes to adopt a challenge-oriented mindset may promote more extraverted and cooperative behaviors (Kavussanu, 2006).

Regarding the mediation findings, threat appraisal was found to reduce prosocial behavior through neuroticism, and to have a similar negative indirect effect through extraversion. These results suggest that the impact of threat perception may vary depending on how individuals internalize it in relation to their psychological characteristics. In particular, neurotic individuals tend to associate threat perception with greater stress, anxiety, and social avoidance. This explains why threat appraisal did not directly affect prosocial behavior in the social context, but rather influenced it indirectly through personality traits (Barlow, 2000; Gross and John, 2003).

The mediating effect of extraversion, on the other hand, points to a different mechanism. Extraversion enables individuals to feel more comfortable in social interactions and makes them more inclined to cooperate with others (Caprara et al., 2012). The present study found that challenge appraisal increased prosocial behavior through extraversion. This finding suggests that perceiving situations as challenges may serve as a direct motivational factor, allowing individuals to build more positive relationships within their social environments (Deci and Ryan, 2000; Dweck, 2012). Specifically, individuals with high levels of challenge appraisal have been shown to develop healthier coping strategies in stressful situations and to seek social support more frequently (Folkman and Lazarus, 1985; Connor-Smith and Flachsbart, 2007).

Although there is no existing study in the literature that directly examines the effects of threat and challenge appraisals on social behaviors along with the mediating role of personality traits, there are several studies that indirectly support the findings of the present research. For example, Chen (2017), in a study exploring the effects of challenge and threat appraisals on stress and emotional processes, showed that challenge appraisal is associated with positive coping strategies, whereas threat appraisal is linked to negative psychological outcomes. This aligns with the current study’s findings, which emphasize the prosocial-enhancing effect of challenge appraisal and the prosocial-reducing effect of threat appraisal. Similarly, Yıldız et al. (2018) investigated the influence of personality traits and moral identity on prosocial and antisocial behaviors in a sports context, and found that extraversion was positively associated with prosocial behavior, while neuroticism was positively related to antisocial behavior. Considering that our study also found extraversion to support social adjustment and neuroticism to be linked to threat perception, these findings further support the mediating function of personality traits. In addition, Cheng et al. (2014) demonstrated in their study on coping strategies that challenge appraisal increases flexible coping, while threat appraisal restricts this process. This finding suggests that threat appraisal may be associated with social withdrawal, whereas challenge appraisal may relate to active social engagement. Finally, Kavussanu and Boardley (2009) showed that threat perception is associated with aggressive and antisocial behaviors, while challenge perception is linked to cooperation and prosocial behaviors. This is consistent with our findings, which highlight how threat and challenge appraisals shape social behaviors through the mediating role of personality traits.

## Conclusion

This study revealed how threat and challenge appraisals influence social behaviors through personality traits, demonstrating that social behaviors are shaped not only by direct cognitive appraisals but also by individuals’ internal dispositions.

For individuals in competitive and high-pressure environments such as athletes developing challenge-oriented thinking strategies is essential for fostering healthier social relationships and promoting prosocial behavior. Previous research in sport psychology has shown that challenge appraisals can enhance individuals’ resilience, motivation, and cooperation within teams (Deci and Ryan, 2000; Kavussanu and Boardley, 2009). In this regard, professional football players’ challenge-focused thinking strategies may serve as a long-term factor in improving social cohesion.

### Strengths, limitations, and recommendations

- This study not only revealed the role of challenge and threat appraisals in explaining individuals’ prosocial and antisocial behaviors, but also comprehensively examined the direct and indirect effects of core personality traits such as extraversion and neuroticism within this relationship. In particular, the path analysis results indicated that challenge appraisal positively predicted prosocial behavior, while threat appraisal increased antisocial behavior. Moreover, the positive indirect effect of extraversion and the negative effect of neuroticism on prosocial behavior significantly contributed to the model. In this regard, the study offers valuable contributions to both the theoretical literature and practical applications in sport psychology.
- The cross-sectional design of the study limits the ability to draw causal conclusions. Additionally, data collection was based solely on self-report instruments, which may increase the risk of social desirability bias. Since the sample was restricted to a specific age group and cultural context, the generalizability of the findings is limited. The fact that only two dimensions (neuroticism and extraversion) of the Five-Factor Personality Model were included should also be considered a methodological limitation.
- Future research may benefit from employing experimental or longitudinal designs to more robustly test causal relationships. Including other personality dimensions (agreeableness, conscientiousness, openness to experience) could provide a more comprehensive understanding of individuals’ social behaviors. The use of observational and multi-source data collection techniques in sport settings would also enhance the validity of future findings.

## Data Availability

The raw data supporting the conclusions of this article will be made available by the authors, without undue reservation.
